# Supplementary material for: Content-rich biological network constructed by mining PubMed abstracts
Source: BMC Bioinformatics. 2004 Oct 8;5:147. doi: 10.1186/1471-2105-5-147 (PMC528731; doi:10.1186/1471-2105-5-147)
Supplement: Additional File 5 — The original Chilibot query results of the term "long-term potentiation (LTP)" and 22 other terms, limiting the latest references analyzed to the years 1990, 1995, 2000, and 2004. [file 1471-2105-5-147-S5.bz2 › chilibotAdditionalFile5/ltp2000/html/ACTIN.html]

 


**ACTIN** (Input: ACTIN ) 

---


**Google Searches:** Entire Web  | EDU domain only  | PDF files only

.

**External Links:** OMIM |
LocusLink
| Swissprot
| GeneCards

  
**Maps of ACTIN**

Simple
Complete
graph in
radiant
tree
square
layout.

**New Hypothesis !**

**Synonyms** 

- actin   [PubMed]

**Synopsis**

- The data obtained support our recent suggestion on the role of filamentous **actin** in induction and long term maintenance of enhanced electrotonic conductivity at mixed synapses.  Tsitologiia, 1999 
  [23]

- Our results strongly suggest that syndapins link endocytosis and **actin** dynamics.  J Cell Biol, 2000 
  [23]

- These results suggest that postsynaptic **actin** filaments are involved in a dynamic process required to maintain AMPAR mediated transmission and to enhance it during LTP.  J Neurosci, 1999 
  [23]

- The results are consistent with a model in which dynamic **actin** filaments play an essential role in the molecular mechanisms underlying the early maintenance phase of LTP, such as growth of new synaptic connections or conversion of silent synapses.  Proc Natl Acad Sci U S A, 2000 
  [20]

- The **actin** binding activity of synapsin I phosphorylated by cAMP dependent protein kinase or by calmodulin dependent protein kinase II showed similar sensitivity to calmodulin inhibition to unphosphorylated synapsin I.  Biochemistry, 1995 
  [19]

- DLS contribution to electrotonic transmission is suggested, direct or mediated by **actin** interaction with nearby localized GJ connexons.  Morfologiia, 1998 
  [19]

- After training, pp60c src from hippocampus showed enhanced interactions with synaptic proteins such as synapsin I, synaptophysin, and the type 2 N methyl d aspartate receptor, as well as the cytoskeletal protein **actin**.  Proc Natl Acad Sci U S A, 2000 
  [16]

- The SH3 mediated interaction of synapsin I with amphiphysins affected the ability of synapsin I to interact with **actin** and synaptic vesicles, and pools of synapsin I and amphiphysin I were shown to associate in isolated nerve terminals.  J Biol Chem, 2000 
  [15]

- While all the anti synapsin I mAbs were unable to affect the interactions of synapsin I both with calcium calmodulin dependent protein kinase II and with **actin** monomers and filaments, mAbs 19.8 and 19.51 were found to inhibit the binding of Grb2 SH3 domains to the proline rich C terminal region of synapsin I.  Brain Res Mol Brain Res, 1997 
  [15]

- The results indicate that the interactions of synapsin II with **actin** are similar   Eur J Neurosci, 1997 
  [14]

- Wehave analyzed by in situ hybridization the distribution of Fx an **actin** sequestering peptide mRNA in the brain of young and old rats.  Neurosci Lett, 1993 
  [14]

- Upon phosphorylation with MAP kinase, synapsin I showed reduced F **actin** bundling activity, while no significant effect on the interaction was observed with the protein phosphorylated with Cdk5 p23.  J Biol Chem, 1996 
  [12]

- These results indicate that the MARCKS protein may modulate release from permeabilized noradrenergic synaptosomes, possibly by modulating calmodulin levels and or the **actin** cytoskeleton.  Neurochem Int, 2000 
  [11]

- When expressed together, the two isoforms form large heterooligomers, and a small fraction of CaMKIIbeta is sufficient to dock the predominant CaMKIIalpha to the **actin** cytoskeleton.  Neuron, 1998 
  [10]

- Furthermore, syndapin I associates with the neural Wiskott Aldrich syndrome protein, an **actin** depolymerizing protein that regulates cytoskeletal rearrangement.  Mol Biol Cell, 1999 
  [10]
